# Medical College Notes

**Published:** 1895-05

**Authors:** 


### Medical College Notes.

The annual meeting of the Alumni Association of the Medical Department of Niagara University will be held at the college building May 16, 1895, one session being in the forenoon and one in the afternoon. An interesting program has been provided, in which the profession is invited to participate. Papers will be read by Dr. Bentley S. Bourne, of Hamburg, N. Y.; Dr. Herman E. Hayd, of Buffalo; and Dr. Joseph M. Mathews, professor of surgery, clinical lecturer on diseases of the rectum, Kentucky School of Medicine, Louisville, Ky.

The commencement exercises of the college will take place the same date at the Academy of Music at 8 o'clock P. M. Prof. Crego will give the valedictory address, and Rev. P. McHale, the new president of Niagara University, will deliver the charge to the graduates.

---

BUFFALO UNIVERSITY MEDICAL COLLEGE announces its annual commencement exercises for Tuesday, April 30, 1895. The final examination before the curators, the Alumni Association meeting and graduating ceremonies occupy the entire day and evening. At the time of this writing (April 23d) the program had not been issued, but a detailed report of the proceedings will be made in the JOURNAL for June.

